# Flavonoids, Isoquinoline Alkaloids, and Their Combinations Affect Growth Performance, Inflammatory Status, and Gut Microbiome of Broilers Under High Stocking Density and Heat Stress

**DOI:** 10.3390/ani15010071

**Published:** 2024-12-31

**Authors:** Kittisak Insawake, Thaweesak Songserm, Ornprapun Songserm, Yongyuth Theapparat, Kazeem D. Adeyemi, Konkawat Rassmidatta, Yuwares Ruangpanit

**Affiliations:** 1 Department of Animal Science, Faculty of Agriculture at Kamphaeng Saen, Kasetsart University, Kamphaeng Saen Campus, Nakhon Pathom 73140, Thailand; kittisak_ku@yahoo.com (K.I.); agropp@ku.ac.th (O.S.); konkawat.dawz@gmail.com (K.R.); 2 Department of Pathology, Faculty of Veterinary Medicine, Kasetsart University, Kamphaeng Saen Campus, Nakhon Pathom 73140, Thailand; fvettss@nontri.ku.ac.th; 3 Center of Excellence in Functional Foods and Gastronomy, Faculty of Agro-Industry, Prince of Songkla University, Hat Yai, Songkhla 90110, Thailand; yongyuth.t@psu.ac.th; 4 Department of Animal Production, Faculty of Agriculture, University of Ilorin, Ilorin 240003, Nigeria; kazyadeyemi@gmail.com

**Keywords:** bacitracin, β-diversity, corticosterone, malondialdehyde, heat stress, stocking density

## Abstract

High stocking density (HSD) and heat stress (HS) challenge broiler production. While antibiotics can mitigate the adverse effects of HS and HSD, their restricted use underscores the need to explore phytochemicals, particularly their combined effects under such conditions. This study investigated the influence of flavonoids, isoquinoline alkaloids, and their combinations as alternatives to bacitracin on growth performance, inflammatory status, gut morphology, and ceca microbiome in broilers raised under HSD and HS. A total of 2100 one-day-old male Ross 308 broiler chicks were distributed into 70 replicates, randomly assigned to one of seven dietary treatments and raised during the summer for 37 days. The treatments included normal stocking density (NSD, 10 birds/m^2^); HSD (15 birds/m^2^); HSD + 50 ppm of bacitracin (BCT); HSD + 300 ppm of flavonoids (FVNs); HSD + 80 ppm of isoquinoline alkaloids (IQAs); HSD + FVNs (1–10 days) and IQAs (11–37 days) (FVN-IQA); and HSD + IQAs (1–10 days) and FVNs (11–37 days) (IQA-FVN). The HS index reached or exceeded 160 during most of the experimental period. From 11 to 24 days of age, the HSD and BCT birds had lower body weight gain. The FVNs, IQAs, and their combinations decreased the corticosterone, IL-6, malondialdehyde, and heterophil–lymphocytes ratio compared to the HSD. Jejunal, ileal, and duodenal villi height/crypt depth ratio was lower in HSD than in other treatments except BCT. The α- and β-diversity, microbiota composition, and metabolic pathways were affected by treatment groups. Overall, FVNs, IQAs, and their combinations improved the growth performance, anti-inflammatory response, and gut health in broilers under HSD and HS, with the combinations exerting synergistic effects.

## Simple Summary

Heat stress (HS) and high stocking density (HSD) in broilers can negatively impact growth performance and gut health. This study investigated the effects of flavonoids, isoquinoline alkaloids, and their combinations as substitutes for bacitracin on growth performance, oxidative stress, immune status, gut morphology, and the cecal microbiome in broilers. Exposure to HSD and heat stress reduced body weight gain and feed efficiency, impaired anti-inflammatory and oxidative stress responses, compromised gut morphology, and disrupted the cecal microbiome. Flavonoids, isoquinoline alkaloids, and their combinations alleviated the detrimental effects of HSD and HS in broilers more effectively than bacitracin, with the combinations exhibiting synergistic benefits.

## 1. Introduction

Due to global warming, heat stress in hot environments has become a global challenge for the poultry industry [1,2]. Heat stress (HS) leads to significant economic losses in the poultry sector, estimated at $240 million annually [3], with summer heat waves resulting in approximately 4 million broiler mortalities [4,5]. Broiler production in tropical regions is particularly impacted by the unique environmental conditions of high temperatures and humidity, which negatively affect production outcomes [6,7]. Chickens are especially vulnerable to heat stress because they lack efficient mechanisms to regulate body temperature, as their feathers provide insulation, and they have few sweat glands [8]. Such stressful conditions impair broiler growth performance, health, and immune responses [8,9]. Increasing bird density is a common strategy to enhance production and lower fixed costs [10]. However, it can elevate the ambient temperature around the birds and thereby exacerbates heat stress [11]. High stocking density (HSD) impairs growth performance, elevates stress markers like the heterophil-to-lymphocyte ratio and corticosterone levels, contributes to oxidative stress, inflammation, apoptosis, and intestinal mucosa damage, and disrupts gut microbiota composition [12,13,14]. The interplay between heat stress and stocking density presents a compounded challenge for poultry production, particularly in tropical regions, where high temperatures and humidity exacerbate the adverse effects of overcrowding [11,15].

Antibiotic growth promoters (AGPs) have been widely used in animal production to enhance growth and reduce morbidity and mortality [16]. However, their use has raised concerns due to the emergence of antimicrobial-resistant bacteria in animals, which pose serious health risks to humans [17]. This situation underscores the urgent need for alternative strategies in animal production. Since 2015, the Department of Livestock Development in Thailand has banned the use of antibiotics as growth promoters in food animals, although their use is still permitted for therapeutic and prophylactic purposes [18]. Over the past four decades, Thailand’s poultry sector has transformed from backyard farming into a leading global exporter, with over 1,000,000 tons of chicken exported in 2023, making it the world’s third-largest chicken exporter [19]. To mitigate the risks associated with antibiotic use and comply with stringent export standards, it is crucial to explore alternative strategies for antibiotic use in poultry production.

Alternatives to antibiotics in animal production include probiotics, prebiotics, organic acids, phytochemicals, and improved management practices such as vaccination, biosecurity, and selective breeding for disease resistance [20,21]. Phytochemicals, naturally derived from plants, present a promising alternative, offering both nutritional and health benefits in livestock production [21,22]. Phytochemicals can alleviate the deleterious effects of HS [23,24] and HSD [25,26] in broilers by reducing oxidative stress and inflammation through their potent antioxidant and anti-inflammatory properties, which lower levels of malondialdehyde, corticosterone, and pro-inflammatory cytokines. In addition, phytochemicals can enhance gut health by modulating the gut microbiota, improve intestinal integrity, and support nutrient absorption, thereby mitigating the damage caused by stress-induced dysbiosis and compromised gut morphology [26,27]. Among them, flavonoids and alkaloids are well known for their antimicrobial, antioxidant, and anti-inflammatory properties, making them valuable for human and animal health [24,28]. One group of alkaloids with medicinal properties is isoquinoline alkaloids (IQAs), derived from *Macleaya cordata*, which contains a mixture of quaternary benzo[c]phenanthridine and protopine alkaloids. IQAs have anti-inflammatory [29] and antioxidant effects [30]. Flavonoids (FVNs), which are secondary metabolites commonly found in plants like citrus fruits, are classified as polyphenols [31] and possess pharmacological properties such as antioxidant [32] and anti-inflammatory effects [33]. They are considered safe and effective in improving animal production efficiency [30,33].

The potential of IQAs to alleviate heat stress [24] and HSD [27], as well as the ability of FVNs to mitigate heat stress [34] and HSD [35] in broilers, has been investigated individually. However, their combined effects have not yet been investigated. In addition, the efficacy of IQAs, FVNs, and their combinations under the simultaneous challenges of heat stress and HSD remains unknown. Due to the antioxidant, immunomodulatory, and antimicrobial properties of IQAs and FVNs, it was hypothesized that their combination will synergistically alleviate the negative impacts of HSD and HS in broilers by reducing oxidative stress, inflammation, and intestinal damage, while improving growth performance and preserving gut microbiota balance. This study investigated the effects of flavonoids, isoquinoline alkaloids, and their combinations as alternatives to bacitracin on the growth performance, oxidative stress, gut morphology, inflammatory status, and ceca microbiome of broilers subjected to HSD and heat stress.

## 2. Material and Methods

### 2.1. Location, Birds, and Experimental Design

The feeding trial was conducted at the Animal Research and Development Center, Faculty of Agriculture at Kamphaeng Saen, Kasetsart University. Kamphaeng Saen is located at 14.0065° N, 99.9889° E. The trial took place during the summer (April to May), with average daytime and nighttime temperatures of 35.83 °C and 26.67 °C, respectively. The average relative humidity during the day and night was 64.55% and 70.2%, respectively.

A total of 2100 one-day-old male broiler chicks (Ross 308) were randomly assigned to seven treatment groups, with 10 replicates per treatment and 30 birds per replicate. According to the National Bureau of Agricultural Commodity and Food Standards [36], the maximum stocking density for broilers is 20 kg/m^2^ or 8 birds/m^2^ in open rearing systems and 33 kg/m^2^ or 14 birds/m^2^ in closed rearing systems. Based on these guidelines, this study utilized a stocking density of 10 birds/m^2^ for the normal stocking condition and 15 birds/m^2^ for the high stocking condition. The concentrations of isoquinoline alkaloids and flavonoids used in this study were determined based on the manufacturer’s specifications and findings from previous research. The experimental treatments were administered until the chicks reached 37 days of age as follows:

(1) Normal stocking density (NSD) with 10 birds/m^2^, fed a basal diet;
(2) High stocking density (HSD) with 15 birds/m^2^, fed a basal diet;
(3) HSD, fed a basal diet supplemented with 50 ppm of bacitracin (BCT);
(4) HSD, fed a basal diet supplemented with 300 ppm of flavonoids (FVNs);
(5) HSD, fed a basal diet supplemented with 80 ppm of isoquinoline alkaloids (IQAs);
(6) HSD, fed a basal diet supplemented with 300 ppm of FVNs (days 1–10) and 80 ppm of IQAs (days 11–37) (FVN-IQA);
(7) HSD, fed a basal diet supplemented with 80 ppm of IQAs (days 1–10) and 300 ppm of FVNs (days 11–37) (IQA-FVN).

The flavonoids used were Bioflavex GC (HealthTech Bio Actives, S.L.U., Barcelona, Spain), and the isoquinoline alkaloids were Sangrovit Extra (Phytobiotics Thailand Co., Ltd., Bangkok, Thailand). Both IQAs and FVNs were tested for purity and composition before use. All birds had ad libitum access to feed and water throughout the experimental period. A lighting schedule of 23 h of light and 1 h of darkness was implemented during the first seven days, followed by 20 h of light and 4 h of darkness until the end of the study. Vaccination was administered as follows: Newcastle disease (ND; B1 strain) and infectious bronchitis (IB) on day 7 (via nasal drops), infectious bursa disease (IBD) on day 14 (via oral drops), and ND (LaSota strain) + IB on day 21 (via nasal drops). The birds were fed corn–soybean meal basal diets formulated according to Ross nutrition specifications [37] (Table 1). The chemical composition of the diets was determined by the AOAC [38] methods and is presented in Table 2.

### 2.2. Heat Stress Index Calculation

Housing temperature and humidity were recorded daily at 7:30 a.m., 12:00 p.m., and 5:30 p.m. The heat stress index was calculated by summing the ambient temperature (in °F) and relative humidity (RH, expressed as a percentage). A heat stress index of 160 or above was considered likely to induce heat stress and negatively affect growth performance [39].

### 2.3. Growth Performance and Blood Parameters

Body weight for each bird in every pen was recorded at days 0, 10, 24, and 37 to calculate body weight gain (BWG). Feed intake and leftover were recorded at days 10, 24, and 37 for each replicate, and average feed intake was calculated periodically. Feed conversion ratio (FCR) was calculated by dividing the total weight of feed consumed by body weight gain. Mortality rates were also recorded to calculate percentage mortality.

At 37 days of age, blood samples were collected from two randomly selected broilers per replicate. Three milliliters of blood were drawn from the wing vein, with 1 mL placed in EDTA tubes for heterophil-to-lymphocyte (H/L) ratio determination. The remaining 2 mL was allowed to clot at 4 °C for 4 h, followed by centrifugation at 3000× *g* for 15 m to collect serum. Serum samples were used to measure corticosterone (CORT) and pro-inflammatory cytokines (IL-1β, IL-6, and TNF-α) using chicken enzyme-linked immunosorbent assay (ELISA) kits (Mybiosource Inc., San Diego, CA, USA). Intra-assay variability was determined by measuring the same sample in triplicate within a single run. The intra-assay coefficient of variation was 3.5%. Lipid peroxidation was assessed via thiobarbituric acid reactive substance (TBARS) assay, quantified as malondialdehyde (MDA) [40].

### 2.4. Detaermination of Intestinal Morphology

At 37 days of age, two birds per replicate were euthanized by CO_2_ inhalation, and samples from the midpoint of duodenum, jejunum, and ileum were collected for histological analysis following the method described by Abudabos et al. [41]. A 2 cm segment of the intestinal sample was fixed in 10% buffered formalin, embedded in paraffin, and sectioned at 5 µm thickness. The sections were stained with hematoxylin and eosin. Measurements of villus height (VH), villus width (VW), and crypt depth (CD) were taken using an Olympus DP22 digital camera and DP2-SAL image analysis system (Olympus Optical Corp., Tokyo, Japan). The VH/CD ratio was calculated as villus height divided by the mucosal crypt depth in each preparation. The SF was measured using the following formula: (2π) × (villus width/2) × (villus length) [42].

### 2.5. Gut Microbiota Using 16S rRNA Sequencing

The quality control of raw sequencing data was performed through processes like read filtering and tag splicing. These steps removed a significant amount of low-quality data, ensuring the accuracy and reliability of subsequent analyses. Genomic DNA was extracted from the ceca samples of two birds per replicate using the ZymoBIOMIC™ DNA Miniprep Kit (Cat. No. D4300, Zymo Research, Tustin, CA, USA) following the manufacturer’s instructions. To prevent bias or erroneous results caused by nucleic acid degradation, samples were collected using DNA/RNA Shield™ (Cat. No. R1100, Zymo Research). DNA quantification was performed with a NanoDrop™ 2000 Spectrophotometer (Thermo Fisher Scientific, Waltham, MA, USA).

For microbial analysis, the V3-V4 region of the 16S rRNA gene was amplified and sequenced using an Illumina NextSeq system (Illumina Inc, San Diego, CA, USA). PCR amplification was conducted using genomic DNA, a forward primer (5′-TCGTCGGCAGCGTCAGATGTGTATAAGAGACAGCCTACGGGAGGCAGCAG-3′), and a reverse primer (5′-GTCTCGTGGGCTCGGAGATGTGTATAAGAGACAGATTACCGCGGCTGCTGG-3′). Each PCR reaction was performed in a 25 µL volume containing Phusion Hot Start II High-Fidelity PCR master mix (Cat. No. F-565S, Thermo Fisher Scientific), 1X Phusion HS II HF Master Mix, 0.2 µM of each primer, and approximately 10 ng of template DNA. Thermal cycling involved an initial denaturation at 95 °C for 3 min, followed by 25 cycles of denaturation at 95 °C for 30 s, annealing at 55 °C for 30 s, and elongation at 72 °C for 30 s, with a final elongation at 72 °C for 5 min. PCR products (~550 bp) were analyzed via 1.5% agarose gel electrophoresis.

DNA quality and concentration were assessed using a QFX Fluorometer (De Novix, Wilmington, DE, USA) and a Bioanalyzer 2100 system (Agilent Technology, Santa Clara, CA, USA). The 16S V3-V4 amplicons were purified from free primers and primer-dimer species using AMPure XP beads (Cat. No. A63881, Beckman Coulter, Indianapolis, IN, USA). The Nextera XT Index Kit was used to attach dual indices and Illumina sequencing adapters, following the manufacturer’s protocol. AMPure XP beads were applied again to clean the final library before quantification. Sequencing libraries were prepared with the NEBNext^®^ Ultra™ II DNA Library Prep Kit for Illumina^®^ (New England Biolabs, Ipswich, MA, USA). After PCR amplicon normalization, 5 µL from each sample well was pooled. The pooled library was quantified using a dsDNA fluorescent dye method with a QFX Fluorometer. Amplicon fragment length was evaluated with the Bioanalyzer 2100 system. Prior to sequencing, amplicons were diluted to 2 nM with resuspension buffer, combined with PhiX Control (2%), and adjusted to a final concentration of 1.5 pM. Indexed primers and sequencing primers were added, and the sequencing library was loaded into an Illumina reagent cartridge. Sequencing was performed on an Illumina MiSeq system (2 × 151 bp paired-end run).

Paired-end sequences from the Illumina MiSeq system were processed using the DADA2 pipeline in QIIME2 (version 2020.11) for data cleaning, denoising, and clustering. The PKSSU 4.0 database served as a reference. Operational taxonomic units (OTUs), alpha diversity, and beta diversity were determined with QIIME2. OTU assignment utilized QIIME’s uclust-based open-reference OTU picking protocol, with the PKSS 4.0 reference set at 99% similarity. Alpha diversity metrics, including Chao1, phylogenetic diversity, and Shannon diversity indices, were calculated. Beta diversity was assessed using weighted UniFrac based on 42,106 randomly selected reads per sample. Principal coordinates analysis (PCoA) was performed to visualize group differences.

The metabolic potential of cecal microbial communities was predicted using 16S rRNA sequences analyzed with PICRUSt2. QIIME2 (version 2019.10) and the q2-picrust2 plugin were employed, with the Kyoto Encyclopedia of Genes and Genomes (KEGG) database used for gene content prediction. KEGG ortholog (KO) genes were annotated by enzyme commission (EC) numbers. Gene counts were grouped by EC numbers and normalized per sample. MetaCyc was used to construct metabolic pathways. Functional outputs were exported to RStudio for further analysis. Statistical analyses and visualizations were performed using the R packages vegan, ggplot2, and STAMP (version 2.1.3). Graphs were created using GraphPad Prism (version 10.4.0).

### 2.6. Statistical Analysis

For the growth performance, blood profile, and gut morphology data, the normality of the distribution and homogeneity of variance were assessed using the Kolmogorov–Smirnov test and the Levene test, respectively. Since the mortality data did not meet the ANOVA assumptions of normality and equal variance, it was subjected to an arcsine square root transformation before analysis. Data were analyzed using one-way ANOVA via the GLM procedure of SAS Studio University Edition (SAS Inst. Inc., Cary, NC, USA). Treatment means were compared using Duncan’s new multiple-range test, with statistical significance set at *p* < 0.05. Alpha diversity was compared across groups with a nonparametric *t*-test with 999 permutations. Beta diversity results were subjected to principal coordinates analysis based on weighted UniFrac followed by permutational multivariate analysis of variance (PERMANOVA) using the Adonis method in R (version 4.2.2) with the vegan package (version 2.5-7). Significant OTUs were identified with negative binomial modeling in DESeq2. A two-sided Welch’s t-test was used for pairwise comparisons of microbial metabolic pathways.

## 3. Results

### 3.1. Heat Stress Index

**Figure 1:**
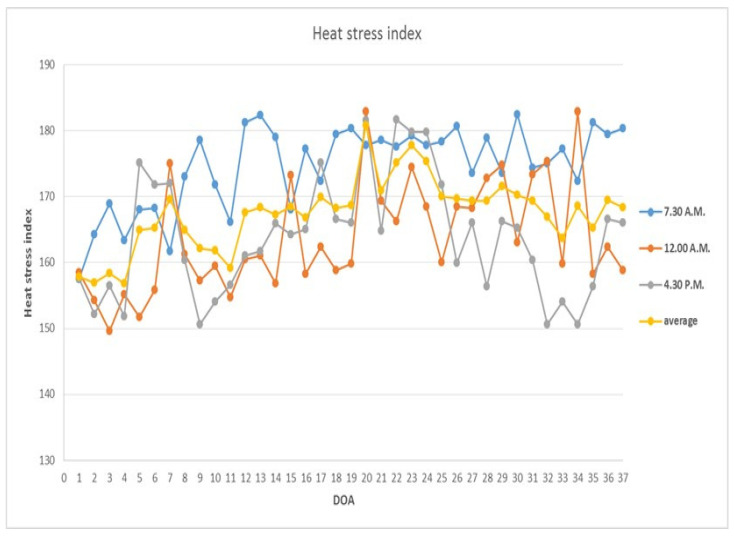
Heat stress index in evaporative cooling system house under tropical conditions. DOA, days of age.

During the experimental period, the heat stress index recorded at 7:30 a.m., 12:00 p.m., and 4:00 p.m. consistently showed values of 160 or higher, which are likely to induce heat stress and negatively impact growth performance (Figure 1).

### 3.2. Growth Performance

In the starter phase (1 to 10 days of age), raising broilers under HSD and heat stress did not significantly affect BWG or FCR compared to NSD (Table 3). However, feed intake was significantly lower (*p* = 0.01) in birds supplemented with FVNs, FVN-IQA, or BCT. During the grower phase (11 to 24 days of age), supplementation with FVNs, IQAs, or their combinations significantly increased BW (*p* = 0.01) in broilers raised under HSD and heat stress. Further, an improvement in FCR was observed (*p* = 0.01) in broilers supplemented with FVN-IQA compared with the HSD and BCT birds. BCT had no significant effect on growth performance during this period. In the finisher phase (25 to 37 days of age), there were no significant differences in growth performance among broilers raised under HSD (*p* > 0.05) (Table 3).

### 3.3. Blood Parameters

Broilers in the HSD group had a significantly higher H/L ratio (*p* = 0.02) compared to those in the NSD group (Table 4). However, supplementation with FVNs, IQAs, and the combination of FVN-IQA or IQA-FVN significantly reduced the H/L ratio and CORT levels (*p* < 0.01) compared to HSD birds. The MDA levels were higher in the HSD group than in the NSD group (*p* < 0.01). FVN and FVN-IQA supplementation significantly lowered MDA levels compared to the HSD group (*p* < 0.01). There were no significant effects of dietary treatments on IL-1β and TNF-α levels. However, FVNs, IQAs, and the combination treatments significantly reduced IL-6 concentration (*p* = 0.03) in comparison to broilers under HSD (Table 4).

### 3.4. Intestinal Morphology

**Figure 2:**
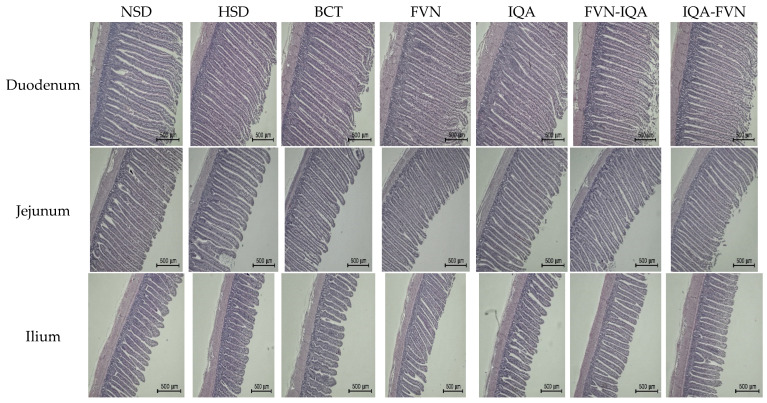
Gut morphology of broilers raised under heat stress and different stocking densities based on hematoxylin and eosin staining. NSD, normal stocking density (10 birds/m^2^) with basal diet; HSD, high stocking density (15 birds/m^2^) with basal diet; BCT, HSD + 50 ppm of bacitracin; FVNs, HSD + 300 ppm of flavonoids; IQAs, HSD + 80 ppm of isoquinoline alkaloids; FVN-IQA, HSD + 300 ppm of FVNs (1–10 days) + 80 ppm of IQAs (11–37 days); IQA-FVN, HSD + 80 ppm of IQAs (1–10 days) + 300 ppm of FVNs (11–37 days).

Figure 2 highlights the gut morphology of broilers under heat stress and HSD. In the duodenum, the HSD group had a lower villus height compared to other treatments (*p* = 0.02) (Table 5). The villus height in the NSD group was similar to that of the supplemented groups, except for the IQA-FVN group. The IQA-FVN group showed a greater villus height than other groups, with the exception of the IQA group. The HSD group had a deeper crypt (*p* = 0.02) compared to other treatments, except for BCT. Crypt depth was similar between the NSD and BCT groups. Villus width did not differ between the HSD group and other groups, except for the IQA-FVN group. The HSD group had a lower VH/CD ratio (*p* = 0.01) compared to other groups, except for BCT, where the VH/CD ratio was not significantly different from that of other groups. Villus surface area in the HSD group did not differ from other groups, except for IQA-FVN and FVN-IQA.

Broilers in the HSD group had a higher CD and lower VH in the jejunum (*p* < 0.01), resulting in a significantly reduced VH/CD ratio (*p* < 0.001) compared to the NSD group (Table 5). However, supplementation with FVNs, IQAs, and the combination treatments significantly increased VH (*p* < 0.05) and decreased CD (*p* < 0.01) compared to the HSD group, leading to a significantly improved VH/CD ratio (*p* < 0.01).

In the ileum, villus height and surface area did not differ between treatment groups (*p* > 0.05). The HSD and BCT groups had deeper crypts compared to other groups (*p* < 0.001). Villus width was similar between the HSD group and other groups. However, villus width was higher in the IQA and FVN groups compared to the NSD, IQA-FVN, and FVN-IQA groups (*p* = 0.01). The HSD group had the lowest VH/CD ratio (*p* < 0.001). The VH/CD ratio in the NSD and BCT groups was similar but lower than that of the IQA, FVN, IQA-FVN, and FVN-IQA groups (Table 5).

### 3.5. Ceca Microbial Diversity

**Figure 3:**
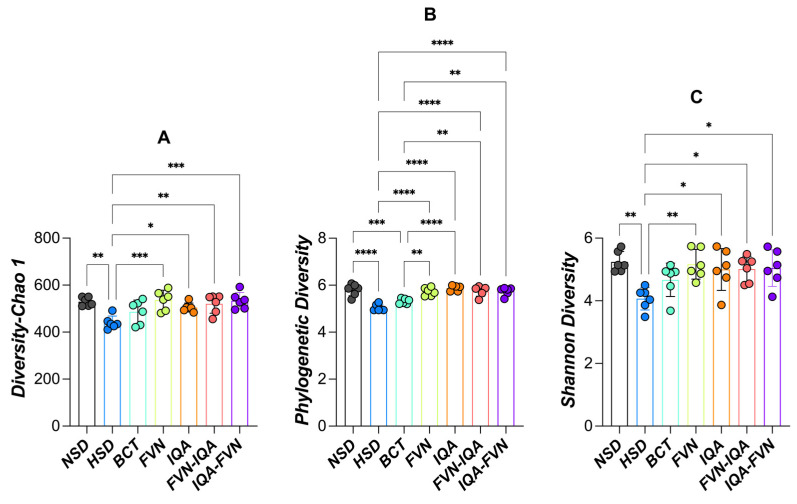
Alpha diversity in terms of Choa1 (**A**), phylogenetic diversity (**B**), and Shannon (**C**) of ceca microbiota of broilers under heat stress and different stocking densities. NSD (black), normal stocking density (10 birds/m^2^) with basal diet; HSD (blue), high stocking density (15 birds/m^2^) with basal diet; BCT (light green), HSD + 50 ppm of bacitracin; FVNs (yellow-green), HSD + 300 ppm of flavonoids; IQAs (orange), HSD + 80 ppm of isoquinoline alkaloids; FVN-IQA (red), HSD + 300 ppm of FVNs (1–10 days) + 80 ppm of IQAs (11–37 days); IQA-FVN (purple), HSD + 80 ppm of IQAs (1–10 days) + 300 ppm of FVNs (11–37 days). * (*p* < 0.05), ** (*p* < 0.01), *** (*p* < 0.001), **** (*p* < 0.0001).

Alpha-diversity analysis using the Chao1, phylogenetic diversity, and Shannon indices revealed significant differences in taxa richness and evenness among treatment groups of broilers raised under heat stress and HSD (*p* < 0.05; Figure 3). Birds in the HSD group exhibited lower Chao1 values compared to other treatments, except for those receiving BCT (Figure 3A). The IQAs, FVNs, and their combinations significantly improved Chao1 values relative to HSD, with the combination treatments showing the highest values. Chao1 values in the NSD group did not differ from other treatments, except for HSD and BCT. Phylogenetic diversity was significantly lower (*p* < 0.05) in the HSD and BCT groups compared to the other treatments (Figure 3B). No significant differences in phylogenetic diversity were observed between the NSD, IQA, FVN, and combination treatment groups. Shannon diversity was also lower (*p* < 0.05) in the HSD group compared to other treatments, except for BCT (Figure 3C). Shannon diversity in the BCT group did not differ significantly from the other treatments.

**Figure 4:**
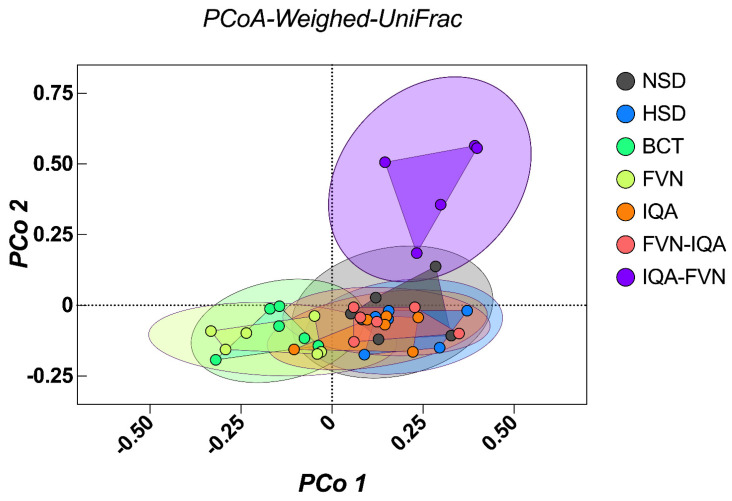
Beta diversity of ceca microbiota of broilers under heat stress and different stocking densities. NSD, normal stocking density (10 birds/m^2^) with basal diet; HSD, high stocking density (15 birds/m^2^) with basal diet; BCT, HSD + 50 ppm of bacitracin; FVNs, HSD + 300 ppm of flavonoids; IQA, HSD + 80 ppm of isoquinoline alkaloids; FVN-IQA, HSD + 300 ppm of FVNs (1–10 days) + 80 ppm of IQAs (11–37 days); IQA-FVN, HSD + 80 ppm of IQAs (1–10 days) + 300 ppm of FVNs (11–37 days).

Beta diversity (Weighted UniFrac distance matrices), as shown by PCoA, indicated distinct clustering between the NSD and HSD groups, highlighting the significant influence of stocking density on microbiota composition (Figure 4). The IQA-FVN combination group formed the most distinct cluster, suggesting a unique microbial profile likely driven by the synergistic effects of the two phytochemicals. The BCT, FVN, and IQA groups showed moderate overlap, indicating some degree of similarity while maintaining distinct microbial profiles. There were significant differences in β-diversity between HSD, NSD, and the supplemented groups (Permannova *p* < 0.05).

### 3.6. Ceca Microbial Composition

**Figure 5:**
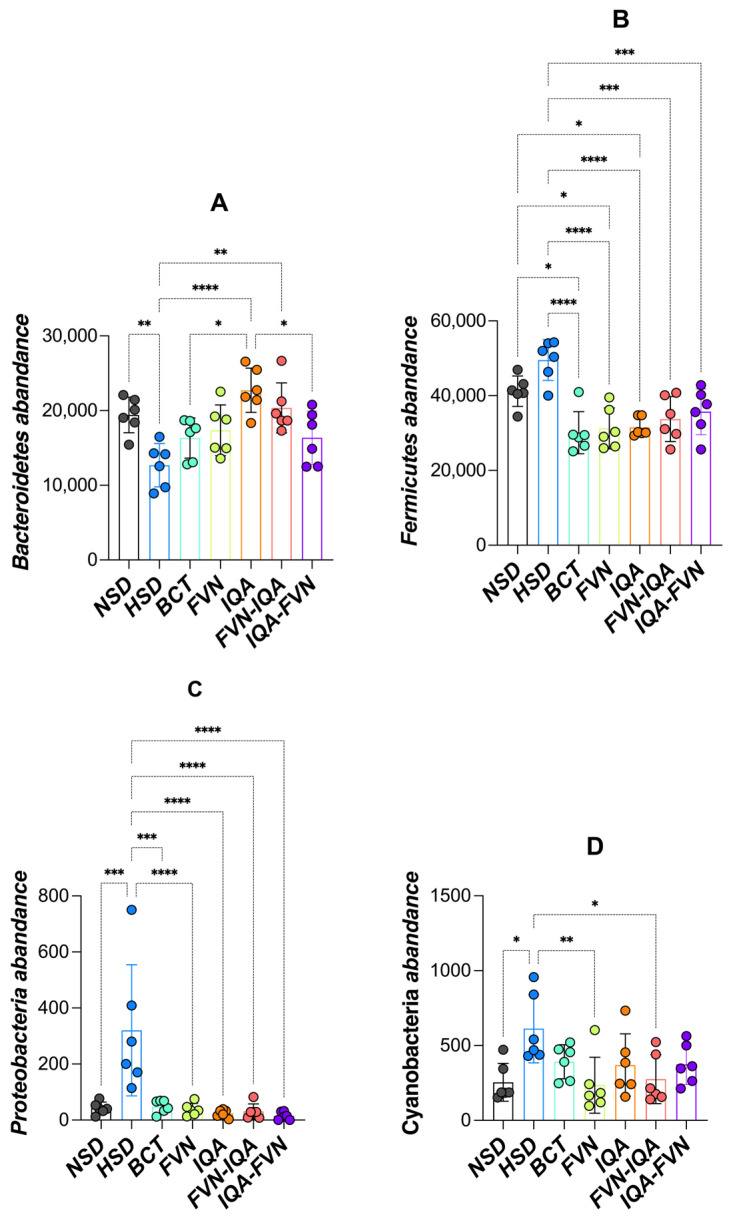
Phylum-level taxonomic distribution of ceca microbiota in broilers under heat stress and different stocking densities. (**A**) Bacteroidetes, (**B**) Firmicutes, (**C**) Proteobacteria, (**D**) Cyanobacteria. NSD (black), normal stocking density (10 birds/m^2^) with basal diet; HSD (blue), high stocking density (15 birds/m^2^) with basal diet; BCT (light green), HSD + 50 ppm of bacitracin; FVNs, (yellow-green) HSD + 300 ppm of flavonoids; IQAs (orange), HSD + 80 ppm of isoquinoline alkaloids; FVN-IQA (red), HSD + 300 ppm of FVNs (1–10 days) + 80 ppm of IQAs (11–37 days); IQA-FVN (purple), HSD + 80 ppm of IQAs (1–10 days) + 300 ppm of FVNs (11–37 days). Bars bearing different superscripts differ significantly. * (*p* < 0.05), ** (*p* < 0.01), *** (*p* < 0.001), **** (*p* < 0.0001).

**Figure 6:**
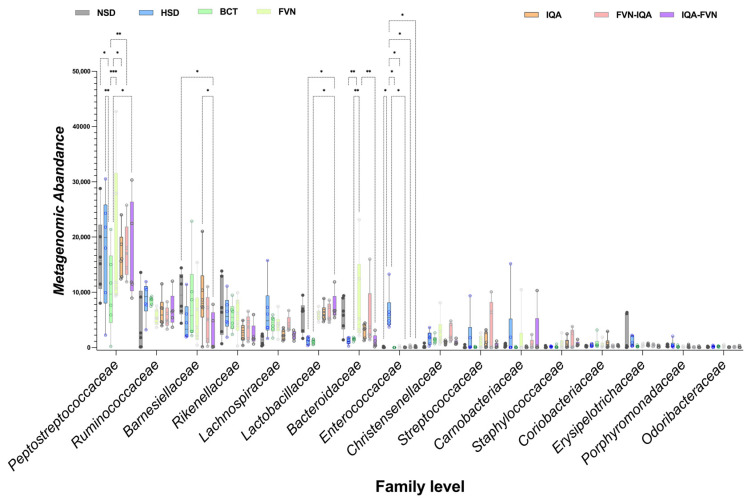
Family-level taxonomic distribution of ceca microbiota in broilers under heat stress and different stocking densities. NSD, normal stocking density (10 birds/m^2^) with basal diet; HSD, high stocking density (15 birds/m^2^) with basal diet; BCT, HSD + 50 ppm of bacitracin; FVNs, HSD + 300 ppm of flavonoids; IQAs, HSD + 80 ppm of isoquinoline alkaloids; FVN-IQA, HSD + 300 ppm of FVNs (1–10 days) + 80 ppm of IQAs (11–37 days); IQA-FVN, HSD + 80 ppm of IQAs (1–10 days) + 300 ppm of FVNs (11–37 days). Bars bearing different superscripts diffesr significantly. * (*p* < 0.05), ** (*p* < 0.01), *** (*p* < 0.001).

**Figure 7:**
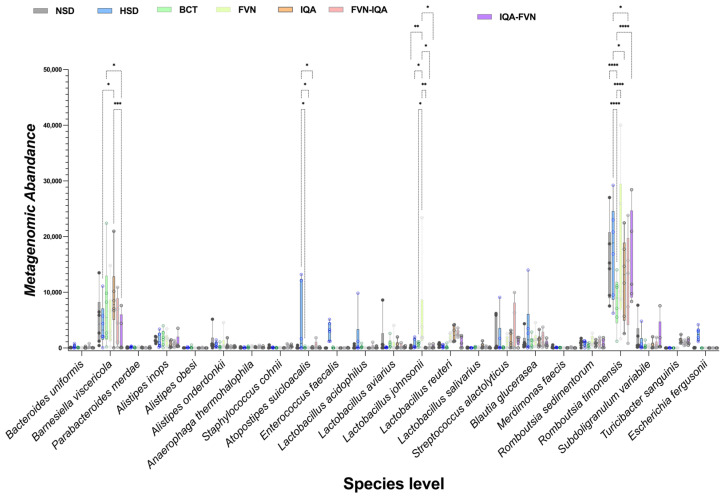
Species-level taxonomic distribution of ceca microbiota in broilers under heat stress and different stocking densities. NSD, normal stocking density (10 birds/m^2^) with basal diet; HSD, high stocking density (15 birds/m^2^) with basal diet; BCT, HSD + 50 ppm of bacitracin; FVNs, HSD + 300 ppm of flavonoids; IQAs, HSD + 80 ppm of isoquinoline alkaloids; FVN-IQA, HSD + 300 ppm of FVNs (1–10 days) + 80 ppm of IQAs (11–37 days); IQA-FVN, HSD + 80 ppm of IQAs (1–10 days) + 300 ppm of FVNs (11–37 days). Bars bearing different superscripts differ significantly. * (*p* < 0.05), ** (*p* < 0.01), *** (*p* < 0.001), **** (*p* < 0.0001).

The relative abundance of microbial phyla (Figure 5), families (Figure 6), and species (Figure 7) in the cecal microbiome of broilers raised under heat stress and HSD was evaluated. Birds in the HSD group exhibited lower levels of Bacteroidetes compared to other treatments (*p* < 0.05) (Figure 5A). The abundance of Bacteroidetes did not differ between the NSD and supplemented groups. The BCT group had lower Bacteroidetes levels than the IQA and FVN-IQA groups. Firmicutes abundance was significantly higher in HSD birds compared to other treatments (*p* < 0.05) (Figure 5B). In contrast, Firmicutes levels in the supplemented groups were lower than those in the NSD group (*p* < 0.05). The abundance of Proteobacteria (Figure 5C) and Cyanobacterium (Figure 5D) was higher in the HSD group compared to all other treatments. The NSD and supplemented groups showed similar levels of Proteobacteria and Cyanobacterium.

At the family level, birds in the HSD and BCT groups had lower abundances of *Lactobacillaceae* and *Bacteroidaceae* (*p* < 0.05) compared to other treatment groups (Figure 6). *Enterococcaceae* abundance was higher in the HSD group relative to the other treatments (*p* < 0.05). *Peptostreptococcaceae* abundance was elevated in the HSD group compared to NSD birds. However, BCT reduced *Peptostreptococcaceae* levels compared to other supplemented groups. FVN supplementation resulted in higher *Peptostreptococcaceae* abundance (*p* < 0.05) than the IQA, FVN-IQA, and IQA-FVN groups. The abundance of *Barnesiellaceae* was lower in the HSD group compared to the NSD, BCT, and IQA groups.

At the species level, HSD birds exhibited lower levels of *Barnesiella viscericola* and higher levels of *Atopostipes suicloacalis*, *Escherichia fergusonii*, and *Enterococcus faecalis* compared to other treatments (*p* < 0.05; Figure 7). FVN supplementation increased the abundance of *Lactobacillus johnsonii* (*p* < 0.05). In addition, the FVN, IQA, FVN-IQA, and IQA-FVN treatments increased the abundance of *Romboutsia timonensis*, while BCT reduced its levels (*p* < 0.05).

### 3.7. Ceca Microbial Metabolic Pathways

**Figure 8:**
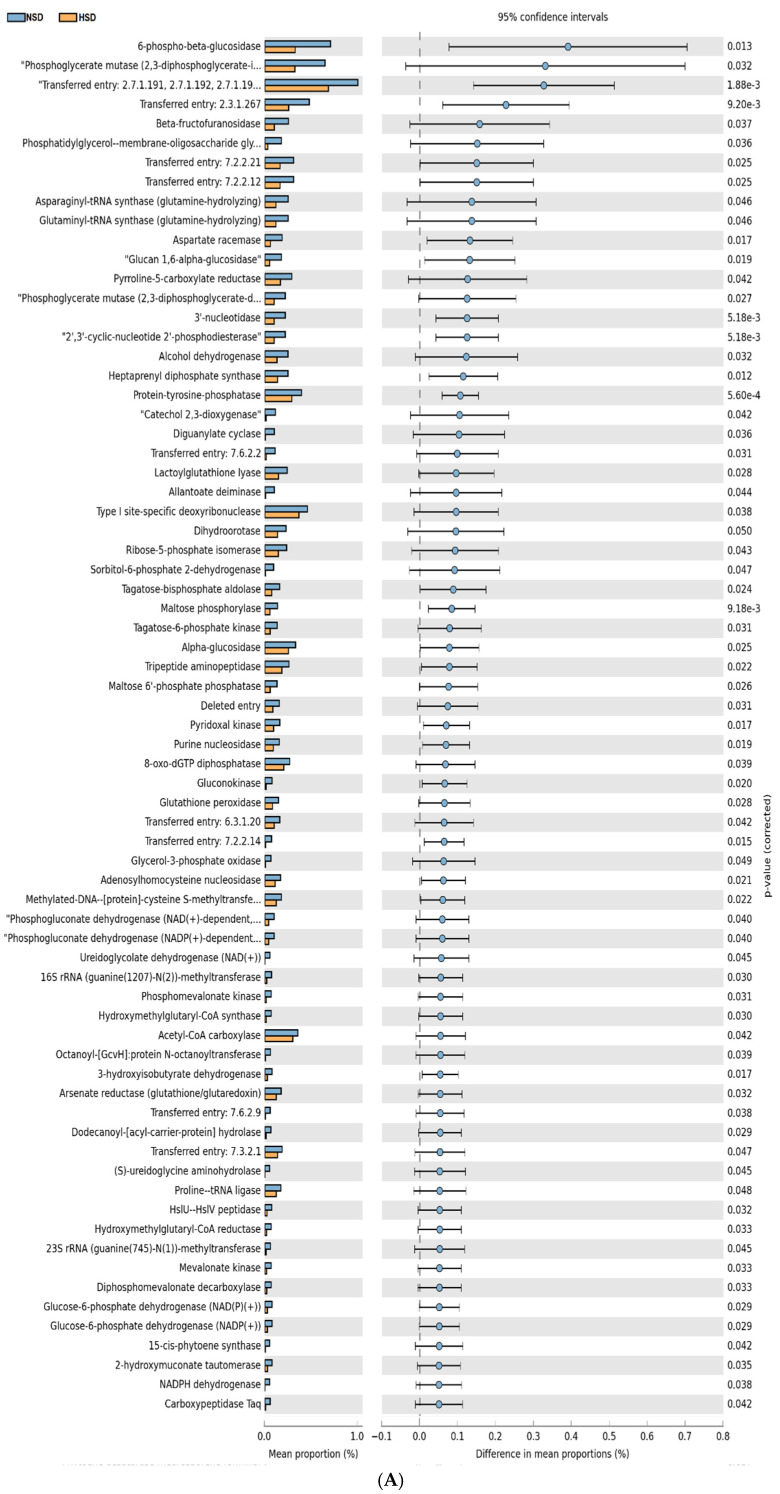

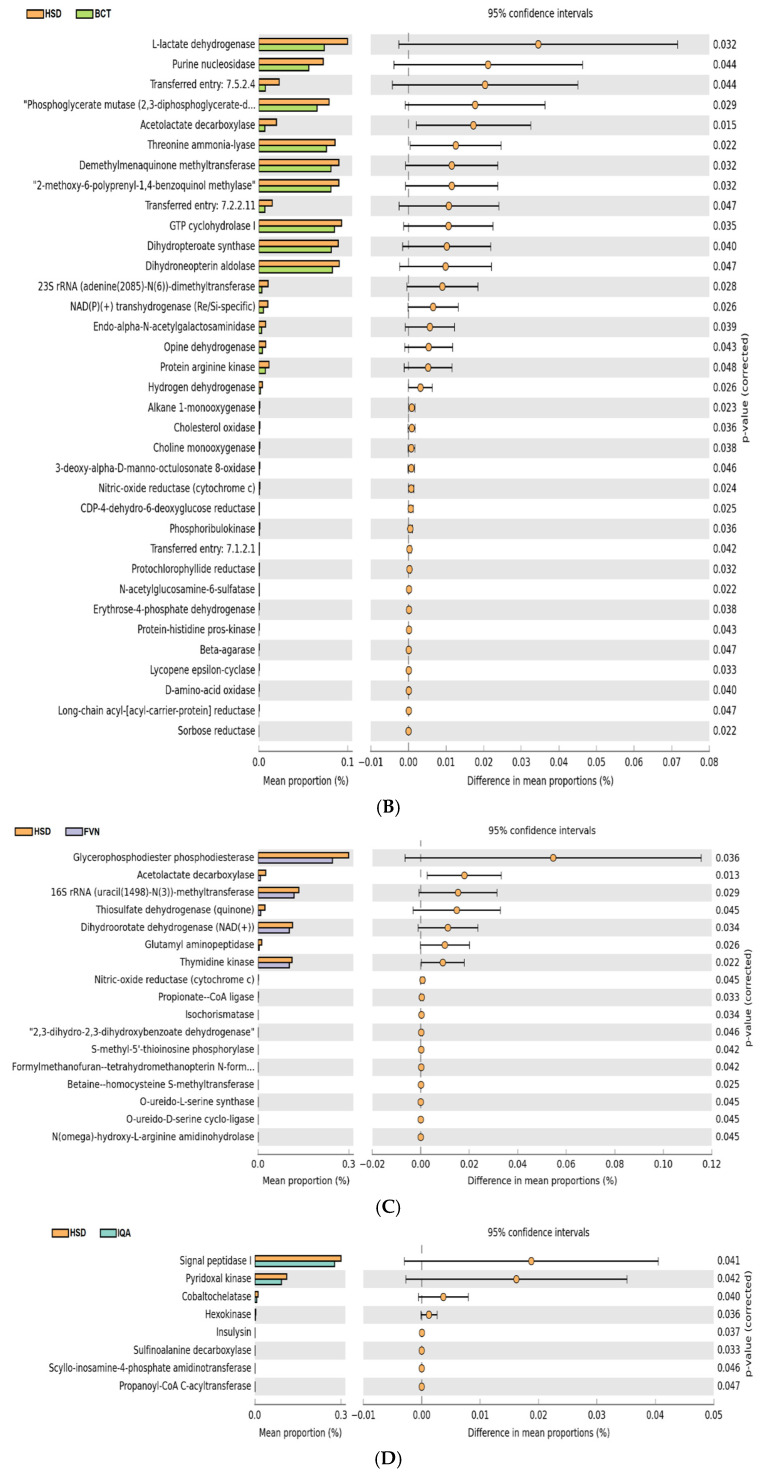

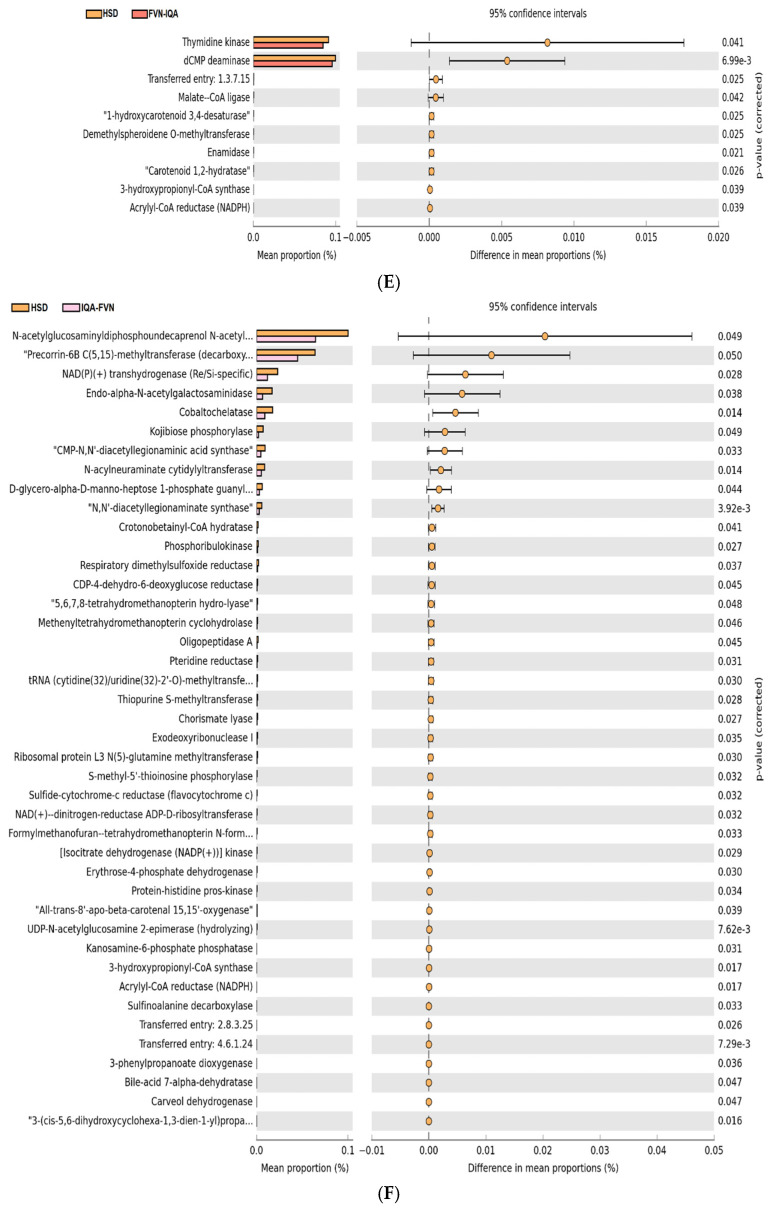
Metabolic pathways of microbial communities in cecal content of broilers predicted from 16S rRNA gene-based microbial compositions using the KEGG annotated databases in HSD vs. NSD (**A**), HSD vs. BCT (**B**), HSD vs. FVNs (**C**), HSD vs. IQAs (**D**), HSD vs. FVN-IQA (**E**), and HSD vs. IQA-FVN (**F**)**.** NSD, normal stocking density (10 birds/m^2^) with basal diet; HSD, high stocking density (15 birds/m^2^) with basal diet; BCT, HSD + 50 ppm of bacitracin; FVNs, HSD + 300 ppm of flavonoids; IQAs, HSD + 80 ppm of isoquinoline alkaloids; FVN-IQA, HSD + 300 ppm of FVNs (1–10 days) + 80 ppm of IQAs (11–37 days); IQA-FVN, HSD + 80 ppm of IQAs (1–10 days) + 300 ppm of FVNs (11–37 days).

The functional profiles of cecal microbiota of broilers under heat stress and HSD revealed 157 metabolic pathways that were significantly altered compared to the NSD group (*p* < 0.05) (Figure 8A). BCT supplementation resulted in the significant expression of 35 metabolic pathways (Figure 8B), while FVN, IQA, FVN-IQA, and IQA-FVN treatments led to the significant expression of 17, 8, 10, and 42 pathways, respectively, compared to the HSD group (Figure 8C–F). Compared to NSD, HSD downregulated several energy-related metabolic pathways, including glucose-6-phosphate dehydrogenase, pyruvate carboxylase, phosphoenolpyruvate, and glycolaldehyde dehydrogenase. Compared to HSD, BCT downregulated pathways such as phosphoglycerate mutase, acetolactate decarboxylase, phosphoribulokinase, and erythrose-4-phosphate dehydrogenase. FVNs, compared to HSD, downregulated acetolactate decarboxylase, propionate-CoA ligase, S-methyl-5′-thioinosine phosphatase, and nitric-oxide reductase (cytochrome C) pathways. IQAs downregulated the pathways for cobaltochelatase, hexokinase, insulysin, and propanoyl-CoA acyltransferase compared to HSD. In the FVN-IQA group, dCMP deaminase, malate-CoA ligase, 3-hydroxypropionyl-CoA synthase, and acrylyl-CoA reductase (NADPH) pathways were downregulated compared to HSD. The IQA-FVN group downregulated kojibiose phosphorylase, crotonobetainyl-CoA hydratase, phosphoribulokinase, and S-methyl-5′-thioinosine pathways compared to HSD.

## 4. Discussion

Broilers raised in tropical climates at high stocking densities often encounter stress due to elevated heat and humidity, which exacerbates the challenges of heat dissipation [43,44]. Throughout much of the experimental period, the heat stress index reached or exceeded 160, which is likely to induce heat stress [39], particularly in Thailand’s summer, where high ambient temperatures and humidity prevail [44]. In such conditions, chickens struggle with cooling down as they primarily rely on panting, a less effective mechanism in humid environments, leading to stress-induced reductions in growth performance [8]. Even with evaporative cooling, the heat stress index surpassed critical thresholds during the day and peaked in the mornings due to the combination of lower temperatures and higher humidity. In addition to the heat stress, HSD intensifies microenvironmental heat, worsening the birds’ stress response [14]. Given chickens’ feathers and lack of sweat glands, they face heightened susceptibility to heat stress [6].

In the starter phase, broilers raised under HSD were less impacted by heat stress due to their smaller size. Therefore, growth metrics such as BWG and FCR remain unaffected. However, as they enter the grower phase and nutritional demands increase, access to feed and water becomes restricted under HSD, leading to slower growth and higher FCR. Stress elevates free radical production, triggering oxidative stress and tissue damage that negatively impact nutrient absorption and growth [45,46]. Consistently, combined HSD and HS decreased growth performance in broilers [11,47]. Supplementation with FVN and IQA antioxidants demonstrated improved body weight and FCR in broilers exposed to both heat and overcrowding stresses. These antioxidants neutralize free radicals, protecting intestinal cells and enhancing nutrient absorption. Broilers supplemented with FVN-IQA showed the best improvement in FCR, likely due to the supplements’ strong cellular antioxidant activity, enhancing free radical scavenging and boosting antioxidant enzymes [28,30]. In the same vein, 4 g/kg propolis supplementation improved growth performance in broilers raised under combined HSD and HS conditions [11].

Physiological markers of stress, including CORT levels and the H/L ratio, provide insight into poultry welfare [48,49]. Stress activates the hypothalamic–pituitary–adrenal axis, releasing hormones that stimulate CORT production [50]. Elevated CORT disrupts lymphocyte levels, increasing heterophil counts and, consequently, the H/L ratio [51]. Both CORT and the H/L ratio were elevated in HSD broilers, indicating stress induced by elevated microenvironmental temperatures [52]. However, the addition of FVNs, IQAs, or a combination of both reduced these stress indicators, reflecting the supplements’ roles in lowering physiological stress levels. Similar reductions in H/L ratios have been observed with genistein and hesperidin supplements [53], and alkaloids from *Rhazya stricta* have also shown similar stress mitigation effects [54].

Oxidative stress, marked by an imbalance between free radical production and antioxidant defenses, leads to cellular damage, with MDA serving as a primary marker [55]. MDA levels were significantly elevated in the HSD broilers, indicative of oxidative damage from combined heat and overcrowding stress [56]. The FVN and FVN-IQA supplementation significantly reduced MDA levels, underscoring their role in maintaining redox balance [28,30]. While bacitracin did not significantly impact MDA levels, flavonoids and alkaloids demonstrated strong antioxidant capabilities, likely explaining their beneficial effects under these conditions.

Heat stress induces inflammation, evidenced by elevated pro-inflammatory cytokines such as IL-1β, IL-6, and TNF-α [7,57]. The oxidative stress caused by high-density conditions intensifies this inflammatory response [58]. Supplementation with FVNs, IQAs, or their combination lowered IL-6 levels, mitigating inflammation under heat stress and HSD. IQAs and FVNs exhibit anti-inflammatory properties [28,30]. Bacitracin also reduced IL-6, consistent with its reported anti-inflammatory effects [59]. The lack of treatment effects on IL-1β and TNF-α levels, in contrast to the significant changes observed in IL-6, may be attributed to differences in the regulatory mechanisms and roles these cytokines play in immune response and inflammation.

The HSD group consistently showed compromised gut morphology, with shorter villi, deeper crypts, and reduced villi height-to-crypt depth ratios, particularly in the duodenum and jejunum. These morphological changes suggest that HSD and HS may induce stress or inflammation in the gut, potentially impairing nutrient absorption and overall gut health [60,61]. Birds under high-stress conditions exhibit reduced blood flow to the intestines, limiting oxygen and nutrient supply, which, in turn, damages the gut lining [62,63]. FVNs, IQAs, and their combination markedly improved gut morphology, particularly villus height and the VH/CD ratio, facilitating better nutrient absorption and overall gut health under stress [64]. Bacitracin, while improving some aspects of gut morphology, was less effective than FVN or IQA supplements.

The lower α-diversity in the HSD groups compared with the other treatments groups except the BCT suggested that both HS and HSD reduce microbial richness and evenness, potentially compromising gut health. This finding is consistent with the elevated corticosterone levels in the HSD birds. High corticosterone alters gut permeability (leaky gut), reduces nutrient absorption, and disrupts the balance of beneficial gut bacteria, thereby lowering microbial richness and evenness. Moreover, heat stress and HSD can induce systemic inflammation and oxidative stress, which can negatively impart commensal bacteria and promotes the growth of opportunistic pathogens, thereby reducing the diversity of beneficial microbes [24,65,66]. Consistent with our findings, HS [65] and HSD [66] reduced α-diversity in broilers. The lower α-diversity in the BCT group may be due to the antimicrobial effect of bacitracin, which selectively suppresses beneficial commensal bacteria, reduces competitive exclusion, and alters gut homeostasis. This may facilitate niche domination by fewer microbial species, ultimately leading to reduced α-diversity. The FVNs, IQAs, and their combinations improved α-diversity with the combination treatments yielding the highest diversity. This may be due to their potential in selectively suppressing pathogens, supporting beneficial microbes, and modulating the gut environment. The combination treatments yielded the highest diversity possibly due to their broader spectrum of action and synergistic effects, fostering a rich and balanced microbial community.

β-diversity indicated by PCoA revealed distinct clustering of the NSD and HSD groups, indicating that stocking density notably influenced gut microflora. HSD groups clustered more closely, with differentiation based on dietary supplementation, while the bacitracin group showed a distinct clustering pattern, likely due to its antimicrobial effects on the microbiome. FVN and IQA supplements also clustered separately, suggesting these compounds’ specific impacts on gut microbiota. The distinct clustering of IQA-FVN suggests a complementary or synergistic effect, which enhances microbial diversity under stress. The potential of IQAs and FVNs to enhance microbial diversity under combined HSD and HS lies in their selective antimicrobial activities, as well as their anti-inflammatory and antioxidant effects, which protect gut integrity and microbial habitats [67]. These compounds also promote mucin production [68], improve gut morphology and nutrient availability, and modulate host gene expression related to immune responses and gut barrier function [6,24], creating a stable environment for diverse microbes [27,65]. In addition, by mitigating stress-related hormonal impacts and supporting short-chain fatty acid-producing bacteria, FVNs and IQAs help maintain a balanced and resilient gut microbiota even under challenging conditions [27,35,65].

Firmicutes and Bacteroidetes dominated the cecal microbiome, consistent with earlier findings [65,69]. The decreased abundance of Bacteroidetes in HSD broilers points to a stressed microbiome state under overcrowding. Bacteroidetes can enhance the host’s ability to utilize polysaccharides [70], boost immunity [71], and help maintain the balance of intestinal microbiota [72]. Through fermentation, they produce short-chain fatty acids, which provide energy and support gut health [73]. Consistently, decreased Bacteroidetes bacteria were found in broilers under HS [65] and HSD [74] conditions. HSD increased the abundance of Firmicutes, Proteobacteria, and Cyanobacterium. An increase in Firmicutes under HSD may indicate dysbiosis, as a high Firmicutes-to-Bacteroidetes ratio is often linked to stress or poor gut health [65,66]. Proteobacteria and Cyanobacterium are often associated with gut inflammation and dysbiosis [65,75]. Their elevated levels under HSD reflect stress-induced gut microbiota imbalance. The BCT, FVNs, IQAs, FVN-IQA, and IQA-FVN restored the abundance of Bacteroidetes, Firmicutes, Proteobacteria, and Cyanobacterium closer to NSD levels, highlighting their role in maintaining gut health under HS and HSD conditions.

The lower abundance of the *Lactobacillaceae* and *Bacteroidaceae* in the HSD birds suggests that HS and HSD negatively affected the beneficial bacterial families associated with gut health and nutrient absorption. Conversely, the increase in the *Enterococcaceae* abundance in the HSD indicated a potential shift towards opportunistic or pathogenic bacteria under stress conditions. A similar disruption in the intestinal microbiota following heat stress [76] and HSD [13] in broilers has been reported. Interestingly, the *Peptostreptococcaceae* family was elevated in the HSD group compared to NSD birds. However, the addition of BCT effectively reduced Peptostreptococcaceae, *Lactobacillaceae*, and *Bacteroidaceae* levels compared to other supplemented groups, suggesting a selective antimicrobial effect of bacitracin. Notably, FVN supplementation led to a higher abundance of *Peptostreptococcaceae* compared to the IQA, FVN-IQA, and IQA-FVN groups, highlighting the differential effects of phytochemical treatments on gut microbiota composition. In addition, *Barnesiellaceae* abundance was lower in the HSD group compared to the other groups, suggesting that the phytochemicals may have helped preserve beneficial bacteria.

HSD lowered the abundance of *Barnesiella viscericola*, a beneficial gut bacterium, while showing elevated levels of *Atopostipes suicloacalis*, *Escherichia fergusonii*, and *Enterococcus faecalis* that are often linked to gut dysbiosis and can negatively impact gut health and performance under stress [64,65,74]. FVN supplementation increased the abundance of *Lactobacillus johnsonii*, a probiotic species known to enhance gut health and immunity. In addition, the FVNs, IQAs, FVN-IQA, and IQA-FVN promoted the growth of *Romboutsia timonensis*, suggesting a positive modulation of gut microbiota. Conversely, BCT supplementation reduced *Romboutsia timonensis* levels, further demonstrating its selective antimicrobial action. Overall, the findings highlight the detrimental effects of HS and HS on beneficial gut microbiota, while phytochemical supplementation contributed to restoring microbial balance and promoting gut health.

Key enzymes in glucose metabolism, glycolysis, and energy production including glucose-6-phosphate dehydrogenase and pyruvate carboxylase were downregulated in HSD broilers, indicating stress-related suppression of energy pathways. This metabolic downregulation aligns with reduced growth rates and nutrient utilization under stress. Bacitracin supplementation further downregulated enzymes involved in glycolysis and amino acid synthesis, possibly due to shifts in gut microbiota, impacting nutrient availability. The FVN supplement shifted energy metabolism away from propionate pathways, while IQA reduced the activity of enzymes related to CoA-dependent metabolism and glucose utilization. Both FVN and IQA supplementation promoted metabolic adjustments that appeared to help broilers adapt more effectively to stress, possibly by balancing energy efficiency and reducing the demand for stress-induced metabolic pathways. Downregulating metabolic pathways like glycolysis and CoA-dependent metabolism in stressed broilers may reduce oxidative stress and inflammation but could also limit energy availability, potentially affecting growth and immune function. However, this trade-off might be beneficial if the reduction in energy-intensive pathways is offset by enhanced nutrient absorption, improved gut health, and a shift towards more efficient energy use. For instance, increasing short-chain fatty acid production could provide alternative energy sources, mitigating the effects of glycolysis downregulation. This metabolic flexibility allows broilers to prioritize gut integrity and immune resilience over rapid growth, ultimately enhancing survival and long-term productivity under stress. These supplements appear to balance these trade-offs, ensuring that growth performance was not compromised while maintaining stress resilience. The combined FVN-IQA treatment downregulated enzymes related to nucleic acid and fatty acid metabolism, suggesting a move toward metabolic efficiency under HSD and heat stress. The IQA-FVN group showed similar effects, specifically on carbohydrate and amino acid metabolism, likely due to improved gut conditions from the combination of antioxidants and anti-inflammatory compounds. These results suggest that combined FVN and IQA supplementation offers a balanced and adaptive metabolic response to crowded, hot environments, promoting improved growth, a balanced gut environment, improved energy utilization, or reduced metabolic stress.

## 5. Conclusions

Under heat stress, HSD in broilers led to notable physiological and morphological challenges, such as increased H/L ratio, CORT and MDA levels, and impaired intestinal morphology compared to those raised under NSD. However, supplementation with FVN, IQA, or their combinations effectively mitigated many of these negative effects, improving growth performance, FCR, and immune stress indicators in broilers under HSD and heat stress. Notably, these supplements helped maintain a more favorable intestinal VH/CD ratio, suggesting enhanced nutrient absorption potential. While BCT showed minimal impact on growth performance, its supplementation influenced the metabolic profile differently from FVNs and IQAs. Heat stress and HSD exert deleterious effects on beneficial gut microbiota, while IQAs, FVNs, and their combinations contributed to restoring microbial balance and promoting gut health. Metabolic pathway analysis revealed that both FVN and IQA treatments downregulated stress-related and energy-draining metabolic pathways, which may have contributed to the observed improvements in growth performance, anti-inflammatory response, and gut health. These findings highlight the potential of FVNs and IQAs, either alone or in combination, as effective alternatives to BCT to alleviate heat stress and HSD-induced stress, improve gut health, and support the overall performance of broilers with the combination exerting synergistic effects. These findings underscore the potential for incorporating the combinations of FVNs and IQAs as effective alternatives to bacitracin in poultry production, offering industry practices a sustainable strategy to mitigate heat stress and high stocking-density-induced challenges, enhance gut health and nutrient absorption, and improve overall broiler performance while aligning with consumer and regulatory demands for reduced antibiotic use. Future studies could focus on testing the synergistic effects of FVN and IQA combinations under various environmental stress conditions or in conjunction with other dietary strategies, as this could provide valuable insights for integrating these alternatives into commercial poultry production systems.

## Figures and Tables

**Table 1:** Ingredients and nutrient composition of basal diets.

Ingredients (% As-Fed Basis)	Starter Diet (D 0–10)	Grower Diet (D 11–24)	Finisher Diet (D 11–37)
Corn	58.28	63.08	64.70
Soybean meal, 48%CP	28.09	21.88	18.42
Full fat soybean	8.0	10.0	12.0
Soybean oil	0.67	0.44	0.72
Mono-dicalcium phosphate (P22)	1.79	1.67	1.55
Limestone	1.46	1.39	1.33
Salt	0.26	0.25	0.24
Broiler vit/min premix ^1^	0.18	0.18	0.18
DL-methionine	0.29	0.25	0.24
L-lysine HCl (96%)	0.23	0.17	0.14
L-threonine	0.19	0.26	0.09
Choline chloride, 60%	0.07	0.07	0.05
Toxin binder	0.15	0.15	0.15
Calculated nutrient composition (% DM)			
Crude protein	22.0	20.0	19.0
Ash	5.72	5.34	5.08
Crude fat	4.79	5.07	5.78
Crude fiber	3.20	3.28	3.36
Calcium	0.96	0.90	0.85
Total phosphorus	0.77	0.73	0.69
Available phosphorus	0.48	0.45	0.42
Choline (mg/kg)	1700	1600	1500
Sodium	0.16	0.16	0.16
Lysine	1.30	1.14	1.07
Methionine	0.56	0.50	0.49
Methionine + cystine	0.90	0.82	0.80
Metabolizable energy (Kcal/kg)	3050	3100	3150

^1^ The composition of vitamins and minerals in 1 kg of premix: vitamin A, 3,000,000 IU; vitamin D3, 600,000 IU; vitamin E, 5000 IU; vitamin K3, 1 g; vitamin B1, 0.5 g; vitamin B2, 1.4 g; vitamin B6, 0.9 g; vitamin B12, 0.5 mg; nicotinic acid, 7 g; folic acid, 0.2 g; biotin, 3 mg; pantothenic acid, 2.21 g; manganese, 12.0 g; zinc, 9.0 g; iron, 16.0 g; copper, 0.32 g; iodine, 80 g; choline chloride, 50 g; selenium, 30 mg.

**Table 2:** Chemical compositions (% DM) of dietary treatments.

Nutrient Components	Control	Bacitracin	Flavonoids	Isoquinoline Alkaloids
Starter diet (d 1–10)				
Moisture %	9.15	9.14	9.08	9.11
Crude protein, %	21.59	21.77	21.59	21.37
Ash, %	6.76	6.85	6.78	6.72
Fat, %	5.08	5.22	5.11	5.26
Fiber; %	1.94	1.90	1.94	1.97
Calcium, %	0.93	0.97	0.95	0.92
Phosphorus, %	0.79	0.78	0.79	0.75
Grower diet (d 11–24)				
Moisture; %	9.30	9.31	9.31	9.32
Crude protein; %	19.12	19.46	19.38	19.28
Ash, %	5.62	5.72	5.73	5.74
Fat, %	6.25	6.28	6.19	6.20
Fiber, %	1.91	1.93	2.03	1.92
Calcium, %	0.91	0.89	0.89	0.90
Phosphorus, %	0.69	0.69	0.69	0.69
Finisher diet (d 25–37)				
Moisture, %	9.36	9.38	9.35	9.35
Crude protein, %	17.95	18.22	17.94	17.90
Ash, %	5.21	5.41	5.43	5.54
Fat, %	6.51	6.54	6.53	6.58
Fiber, %	1.96	2.01	1.94	1.93
Calcium, %	0.87	0.84	0.83	0.88
Phosphorus, %	0.59	0.60	0.60	0.59

**Table 3:** Effect of dietary bacitracin, flavonoids, and isoquinoline alkaloids on growth performance in broilers under heat stress and different stocking densities ^1^.

Parameter	Treatment ^2^							Pooled SE	*p* Value
	NSD	HSD	BCT	FVN	IQA	FVN-IQA	IQA-FVN		
Initial BW (g/bird)	41.23	41.18	41.14	41.21	41.19	41.20	41.18	0.049	0.99
1–10 days									
Feed intake (g/bird)	289.2 ^a^	289.8 ^a^	283.8 ^b^	287.4 ^ab^	288.6 ^a^	284.1 ^b^	289.8 ^a^	0.574	0.01
BWG (g/bird)	247.7	245.6	247.4	246.8	248.1	245.1	249.0	0.596	0.60
FCR	1.17	1.18	1.15	1.17	1.16	1.16	1.16	0.003	0.06
Mortality (%)	0.0	0.0	0.0	0.0	0.0	0.0	0.0	N/A	N/A
11–24 days									
Feed intake (g/bird)	1052	1063	1059	1071	1079	1047	1071	3.872	0.30
BWG (g/bird)	722.9 ^a^	704.3 ^b^	702.9 ^b^	724.1 ^a^	721.5 ^a^	718.0 ^a^	721.8 ^a^	1.834	0.01
FCR	1.46 ^b^	1.51 ^a^	1.51 ^a^	1.48 ^ab^	1.49 ^a^	1.46 ^b^	1.48 ^ab^	0.005	0.01
Mortality (%)	0.91	1.25	1.56	0.94	1.56	1.25	1.25	0.249	0.99
25–37 days									
Feed intake (g/bird)	1887	1859	1899	1891	1878	1839	1882	9.366	0.65
BWG (g/bird)	1113	1093	1131	1110	1112	1104	1127	6.766	0.86
FCR	1.70	1.71	1.68	1.71	1.69	1.67	1.68	0.010	0.94
Mortality (%)	0.00	1.27	0.32	1.59	0.00	0.64	0.00	0.18	0.08
1–37 days									
Feed intake (g/bird)	3228	3212	3243	3249	3246	3170	3243	11.98	0.57
BWG (g/bird)	2084	2043	2081	2081	2081	2067	2160	7.670	0.70
FCR	1.55	1.57	1.56	1.56	1.55	1.53	1.55	0.006	0.69
Mortality (%)	0.91	2.50	1.88	1.88	1.56	1.88	1.25	0.324	0.90

^a,b^ Means bearing different superscripts in a row differ significantly (*p* < 0.05). ^1^ Each value represents the mean of 10 replicates. ^2^ NSD, normal stocking density (10 birds/m^2^) with basal diet; HSD, high stocking density (15 birds/m^2^) with basal diet; BCT, HSD + 50 ppm of bacitracin; FVNs, HSD + 300 ppm of flavonoids; IQAs, HSD + 80 ppm of isoquinoline alkaloids; FVN-IQA, HSD + 300 ppm of FVNs (1–10 days) + 80 ppm of IQAs (11–37 days); IQA-FVN, HSD + 80 ppm of IQAs (1–10 days) + 300 ppm of FVNs (11–37 days). BW, body weight; BWG, body weight gain; FCR, feed conversion ratio.

**Table 4:** Effect of dietary bacitracin, flavonoids, and isoquinoline alkaloids on serum oxidative and immune indices in broilers under heat stress and high stocking density ^1^.

Parameter	Treatment ^2^							Pooled SE	*p* Value
	NSD	HSD	BCT	FVN	IQA	FVN-IQA	IQA-FVN		
H/L ratio	1.926 ^b^	2.427 ^a^	2.135 ^ab^	1.904 ^b^	1.995 ^b^	1.867 ^b^	1.989 ^b^	0.009	0.02
Corticosterone (ng/mL)	25.87 ^b^	30.91 ^a^	25.06 ^bc^	25.03 ^bc^	25.14 ^bc^	21.34 ^c^	22.55 ^bc^	0.010	<0.001
Malondialdehyde (μM/100 μL)	10.28 ^b^	11.47 ^a^	11.38 ^a^	10.59 ^b^	10.89 ^ab^	10.69 ^b^	10.88 ^ab^	0.003	<0.01
IL-1β (pg/mL)	206.1	214.9	211.6	210.2	204.2	204.2	207.0	0.005	0.89
IL-6 (pg/mL)	285.8 ^ab^	310.8 ^a^	277.2 ^ab^	266.3 ^b^	264.0 ^b^	252.5 ^b^	265.3 ^b^	0.007	0.03
TNF-α (ng/mL)	41.14	46.23	46.84	41.76	40.25	39.37	41.57	0.013	0.39

^a,b,c^ Means bearing different superscripts in a row differ significantly (*p* < 0.05). ^1^ Each value represents the mean of 10 replicates. ^2^ NSD, normal stocking density (10 birds/m^2^) with basal diet; HSD, high stocking density (15 birds/m^2^) with basal diet; BCT, HSD + 50 ppm of bacitracin; FVNs, HSD + 300 ppm of flavonoids; IQAs, HSD + 80 ppm of isoquinoline alkaloids; FVN-IQA, HSD + 300 ppm of FVNs (1–10 days) + 80 ppm of IQAs (11–37 days); IQA-FVN, HSD + 80 ppm of IQAs (1–10 days) + 300 ppm of FVNs (11–37 days).

**Table 5:** Effect of dietary bacitracin, flavonoids, and isoquinoline alkaloids on gut morphology in broilers under heat stress and different stocking densities ^1^.

Parameter	Treatment ^2^							Pooled SE	*p* Value
	NSD	HSD	BCT	FVN	IQA	FVN-IQA	IQA-FVN		
Duodenum									
Villi height (µm)	1731.0 ^bc^	1663.6 ^d^	1726.9 ^c^	1751.2 ^bc^	1780.7 ^b^	1809.0 ^ab^	1869.6 ^a^	123.3	0.02
Crypt depth (µm)	184.0 ^b^	208.9 ^a^	194.3 ^ab^	182.0 ^c^	184.4 ^c^	194.4 ^b^	194.2 ^b^	18.42	0.02
Villi width (µm)	137.3 ^c^	145.2 ^bc^	150.4 ^bc^	153.3 ^b^	152.5 ^b^	156.1 ^ab^	160.6 ^a^	11.15	<0.001
Villi height/crypt depth ratio	9.86 ^a^	8.72 ^b^	9.41 ^ab^	9.85 ^a^	9.96 ^a^	9.57 ^a^	9.55 ^a^	0.780	0.01
Villi surface area (mm^2^)	0.75 ^c^	0.79 ^bc^	0.82 ^bc^	0.84 ^b^	0.86 ^ab^	0.89 ^a^	0.94 ^a^	0.107	0.01
Jejunum									
Villi height (µm)	1176 ^a^	1074 ^b^	1142 ^ab^	1143 ^ab^	1179 ^a^	1199 ^a^	1183 ^a^	106.6	<0.01
Crypt depth (µm)	153.0 ^b^	162.0 ^a^	164.1 ^a^	155.2 ^b^	147.2 ^c^	142.0 ^c^	1367.0 ^c^	16.52	<0.01
Villi width (µm)	136.3 ^b^	142.3 ^a^	138.6 ^ab^	135.5 ^b^	132.4 ^b^	129.7 ^c^	129.3 ^c^	8.69	0.01
Villi height/crypt depth ratio	7.95 ^b^	6.87 ^c^	7.12 ^bc^	7.54 ^b^	8.11 ^b^	8.66 ^a^	9.15 ^a^	0.664	<0.001
Villi surface area (mm^2^)	0.504	0.483	0.499	0.489	0.485	0.493	0.493	0.062	0.99
Ileum									
Villi height (µm)	862.5	821.4	845.0	873.8	892.1	870.4	866.7	93.44	0.74
Crypt depth (µm)	153.5 ^b^	171.8 ^a^	158.8 ^a^	137.5 ^b^	141.0 ^b^	132.2 ^b^	142.3 ^b^	18.30	<0.001
Villi width (µm)	113.1 ^b^	119.8 ^ab^	119.9 ^ab^	123.3 ^a^	121.3 ^a^	114.2 ^b^	111.3 ^b^	7.925	0.01
Villi height/crypt depth ratio	5.89 ^b^	5.01 ^c^	5.53 ^b^	6.61 ^a^	6.51 ^a^	6.84 ^a^	6.29 ^a^	0.584	<0.001
Villi surface area (mm^2^)	0.307	0.294	0.318	0.336	0.338	0.310	0.303	0.043	0.19

^a,b,c^ Means bearing different superscripts in a row differ significantly (*p* < 0.05). ^1^ Each value represents the mean of 10 replicates. ^2^ NSD, normal stocking density (10 birds/m^2^) with basal diet; HSD, high stocking density (15 birds/m^2^) with basal diet; BCT, HSD + 50 ppm of bacitracin; FVNs, HSD + 300 ppm of flavonoids; IQAs, HSD + 80 ppm of isoquinoline alkaloids; FVN-IQA, HSD + 300 ppm of FVNs (1–10 days) + 80 ppm of IQAs (11–37 days); IQA-FVN, HSD + 80 ppm of IQAs (1–10 days) + 300 ppm of FVNs (11–37 days).

## Data Availability

The raw data supporting the conclusions of this article will be made available by the corresponding author on request.
